# Impact of Non-Human Leukocyte Antigen-Specific Antibodies in Kidney and Heart Transplantation

**DOI:** 10.3389/fimmu.2017.00434

**Published:** 2017-04-13

**Authors:** Xiaohai Zhang, Nancy L. Reinsmoen

**Affiliations:** ^1^HLA and Immunogenetics Laboratory, Comprehensive Transplant Center, Cedars-Sinai Medical Center, Los Angeles, CA, USA

**Keywords:** human leukocyte antigen, non-human leukocyte antigen antibody, kidney transplant, heart transplant, angiotensin II type 1 receptor antibody

## Abstract

The presence of donor human leukocyte antigen (HLA)-specific antibodies has been shown to be associated with graft loss and decreased patient survival, but it is not uncommon that donor-specific HLA antibodies are absent in patients with biopsy-proven antibody-mediated rejection. In this review, we focus on the latest findings on antibodies against non-HLA antigens in kidney and heart transplantation. These non-HLA antigens include myosin, vimentin, Kα1 tubulin, collagen, and angiotensin II type 1 receptor. It is suggested that the detrimental effects of HLA antibodies and non-HLA antibodies synergize together to impact graft outcome. Injury of graft by HLA antibodies can cause the exposure of neo-antigens which in turn stimulate the production of antibodies against non-HLA antigens. On the other hand, the presence of non-HLA antibodies may increase the risk for a patient to develop HLA-specific antibodies. These findings indicate it is imperative to stratify the patient’s immunologic risk by assessing both HLA and non-HLA antibodies.

## Introduction

Despite the advancement of improved immunosuppression regimens and optimized patient management, antibody-mediated rejection remains a major obstacle for long-term graft survival (1). Initial emphasis has been on the identification of human leukocyte antigen (HLA)-specific antibodies directed against the donor HLA class I and/or class II antigens. With the introduction of commercially available solid phase assay reagents, a greater understanding of the specificity, strength, and function of these antibodies has been made possible. Further, the wide acceptance of these reagents in the clinical transplant testing setting, along with proficiency testing programs has allowed for increased consistency in the definition of the antibodies detected. Correlating the antibody information obtained together with the clinical graft outcome has revealed that patients with antibodies against donor HLA are at a higher risk of antibody-mediated rejection and poorer graft outcome (2, 3). However, for many patients who have allograft dysfunction and show histological characteristics of antibody-mediated rejection on biopsy, no donor HLA-specific antibodies are detected (2, 4). In heart transplant, it has been shown that 40% of patients diagnosed with biopsy-proven antibody-mediated rejection did not show donor HLA-specific antibodies in the peripheral blood (5). These studies initiated investigation into non-HLA-specific antigens, many of which are expressed by the vascular endothelium and often revealed after stress or graft injury. Even though the detection of many of these non-HLA antibodies remains elusive, antibodies to the following non-HLA antigens have been shown to be associated with graft dysfunction or rejection: major histocompatibility complex class I chain-related gene A (MICA), angiotensin II type 1 receptor (AT_1_R), endothelin type A receptor (ET_A_R), heat shock protein, phospholipid, K-α-tubulin, vimentin, and endothelial cell antigens (6). Recently, commercial reagents have become available to define antibodies to AT_1_R, ET_A_R, MICA, and endothelial cell antigens. These results have been used in the clinical testing setting to investigate their impact on graft outcome. Studies of antibodies to other non-HLA antigens have been largely single center results. The results for anti-HLA and non-HLA-specific antibodies indicate an interplay between alloimmunity and autoimmunity, impacting graft outcome. This review focuses on the contribution on antibodies against these non-HLA antigens in kidney and heart transplantation. For lung transplant outcomes, please refer to the comprehensive review by Dr. Mohanakumar in this same issue.

## Antibodies to MICA

Major histocompatibility complex class I chain-related gene A gene is localized in the HLA gene cluster. The MICA protein shares similar structure with HLA class I but does not associate with β2 microglobulin at the cell surface and cannot bind peptides. MICA can activate natural killer (NK) cells *via* interaction with activating immunoreceptor NKG2D (7). Of the non-HLA antigens listed above, MICA is highly polymorphic with around 100 alleles identified as of July 2016. Similar to HLA molecules, the recipient and donor may carry different MICA alleles. The recipient’s immune system may develop antibodies against the donor-specific MICA allele (8). It has been reported that 5–9% of renal recipients display MICA antibodies (9). The contribution of MICA antibody to pathogenesis of antibody-mediated rejection was first demonstrated in kidney transplantation (10) and was later found to be associated with rejection in pancreas and heart transplant (11, 12). Further, the patient with antibodies against donor-specific MICA is at higher risk of antibody-mediated rejection (5). The expression of MICA is not detectable on the quiescent endothelial cells which lie at the interface of the allograft and recipient blood and are directly targeted by immune response. The expression of MICA can be induced by stress and cytokines, such as TNF-α (13). The development of MICA antibodies may indicate an underlying inflammatory status which exists in these conditions.

Studies have demonstrated that expression of MICA in tumor cells leads to the activation of NK cells *via* MICA/NKG2D interaction, which in turn release cytotoxic proteins and INF-γ (7). Binding of MICA antibody on endothelial cells may block interaction between MICA and NKG2D, and thereby dampen NKG2D-mediated cytotoxicity. However, NK cells may still be activated mainly through Fc receptor-dependent cytotoxicity.

### Antibodies against G Protein-Coupled Receptors (GPCRs)

AT_1_R and ET_A_R belong to the family of GPCRs which have seven transmembrane domains. Antibodies to the GPCRs, AT_1_R, and ET_A_R, may be relevant due to their endothelial cell surface expression and extracellular regions accessible to antibodies. Some of these antibodies, such as those to AT_1_R, have been shown to be involved in the pathophysiology of pregnancy preeclampsia and autoimmune diseases, including systemic sclerosis (14–16). There are several possible mechanisms relevant to explain how patients without an autoimmune disease may develop these autoantibodies. One plausible reason is the immune suppression or an underlying inflammatory process may break the self-tolerance. Also, a shearing process induced by mechanical circulatory systems or dialysis may cause proteins, such as von Willebrand factor, cleaved into smaller peptides (17). It is possible that the extracellular loop of AT_1_R may be clipped off the cell surface by shear stress and thereby exposing a neoantigen. The cell surface density of these GPCRs is influenced by polymorphisms and different mRNA processing mechanisms. The severity of injury by AT_1_R antibodies may also be influenced by the expression level of these different AT_1_R isotypes on the allograft. These antibodies may not only target the allograft but may also have a global effect. The impact of anti-AT_1_R antibodies in a clinical setting was first identified in a group of kidney-transplant recipients with malignant hypertension (18), suggesting that binding of AT_1_R antibodies, similar to the ligation of AT_1_R with angiotensin II, can also promote vasoconstriction, water intake, and sodium retention and increase blood pressure (19). Similar to HLA antibodies, AT_1_R antibodies may have detrimental effect on the graft survival. The presence of AT_1_R antibodies is associated with antibody-mediated rejection, but not cellular-mediated rejection in kidney transplant (20). In heart transplant, increased levels of AT_1_R have been shown to be associated with antibody-mediated rejection, cellular-mediated rejection, and early onset of microvasculopathy at 1 year posttransplant (21). In the same study, high levels of antibodies against another GPCR, ET_A_R, also has been reported to be associated with antibody or cell-mediated rejection. The associations observed with either antibody-mediated rejection or cellular rejection are dependent on the current pathological definition of these types of rejection in each organ group.

AT_1_R antibodies can synergize with HLA antibodies to predispose the graft to rejection. The presence of both strong binding AT_1_R antibody and HLA class II donor-specific antibodies has been found to be associated with accelerated rejection, hypertensive encephalopathy, and worse graft survival in kidney transplant (20, 22, 23). In the absence of donor-specific HLA or MICA antibodies, strong binding AT_1_R antibodies have been detected in patients with antibody-mediated rejection. Furthermore, transplant candidates with strong AT_1_R antibodies pretransplant are at a higher risk of early antibody-mediated rejection and long-term graft loss.

Activation of AT_1_R by its native ligand angiotensin II stimulates phospholipase C, production of cyclic adenosine monophosphate, and activation of mitogen-activated protein kinase, which promotes vasoconstriction and hypertension (24). Association between AT_1_R antibody and rejection was first reported in patients with malignant hypertension by Dragun and her colleagues. They found that AT_1_R antibodies bind to the extracellular loop 2 of AT_1_R and stimulate phosphorylation of extracellular signal-regulated kinase (ERK) (18). However, studies indicate that the angiotensin II binding pocket of AT_1_R is composed of all seven transmembrane helices and both extracellular loops 1 and 2 (25, 26), which is distinct from the antibody-binding site. It is not clear how AT_1_R antibody stimulates similar signals as native ligand angiotensin II. Several other studies have shown the association of AT_1_R antibodies with rejection, but none of these studies reported hypertension in their cohort or if these antibodies could activate signaling to stimulate hypertension (20, 21, 27–30). AT_1_R antibodies have been found to be the IgG1 and IgG3 subclasses which have the capacity to activate the complement cascade (18). Interestingly, detrimental effect of AT_1_R antibodies on the graft does not need complement activation. Reinsmoen et al. observed C4d-positive biopsy in only one out of six patients who had strong AT_1_R antibodies and were diagnosed as antibody-mediated rejection (20). In consistent with this observation, Fuss et al. reported that AT_1_R antibodies, but not donor-specific HLA antibodies, were detected in 11 cases of biopsy-proven, C4d-negative acute antibody-mediated rejection according to Banff 2013 (29). Nevertheless, AT_1_R antibodies may still be detrimental to the allograft through antibody-dependent cell-mediated cytotoxicity.

AT_1_R also plays an important role in glucose metabolism (31). It is suggested that higher expression of AT_1_R is associated with increased risk for diabetic nephropathy, and blockade of AT_1_R signaling is effective in the treatment of diabetic nephropathy (31, 32). AT_1_R antibody can also activate AT_1_R signaling. The presence of AT_1_R antibody in renal transplant candidate might predispose development of diabetic nephropathy. Further study on the role of AT_1_R antibody in diabetic nephropathy is warranted.

A commercially available test reagent provides detection of AT_1_R antibody in a relatively easier and standardized manner compared to other non-HLA antibodies. However, this assay is enzyme-linked immunosorbent assay based and is sensitive to interference by intravenous immunoglobulin and rituximab treatment. The linear range of results obtained from this assay is also rather narrow. Improvement of the AT_1_R-testing reagent will promote more transplant programs to adopt the AT_1_R antibody test.

## Antibodies to Vimentin

Vimentin is a subunit of an intermediate filament. As a cytoskeletal element, vimentin is important for stabilizing the architecture of the cytoplasm. However, vimentin is also found to be secreted by macrophages, endothelial cells, vascular smooth muscle cells, activated platelets, apoptotic T cells, and neutrophils (33). Secretion is increased by the pro-inflammatory cytokine TNF-α, but inhibited by the anti-inflammatory cytokine IL10, suggesting that vimentin may be involved in the immune response (34). Antibodies against citrullinated vimentin have been detected in sera of patients with rheumatoid arthritis (35). Vimentin antibodies also exist in pretransplant sera of patients with end-stage kidney disease (36). In this study, titers of IgM antibodies against vimentin increased every year posttransplant compared with pretransplant titers, but no difference was found in patients with interstitial fibrosis and tubular atrophy compared with a kidney recipient control group. By contrast, patients diagnosed with interstitial fibrosis and tubular atrophy have higher levels of IgG antibodies against vimentin. These results suggest that IgG, but not IgM, antibodies against vimentin may play a role in the pathology of interstitial fibrosis and tubular atrophy (37). Similarly, in heart transplant, patients with vimentin antibodies within the first 2 years of transplantation are at higher risk for cardiac allograft vasculopathy at 5 years posttransplant (38). The presence of vimentin antibodies is rather common in heart transplant recipients. Young et al. have shown 34% of patients display vimentin antibodies, but no difference in the rates of early rejection and graft survival was observed (39).

## Anti-Myosin Antibodies

Myosins are a large family of proteins which bind actin cytoskeleton and move cargo proteins through ATP hydrolysis. The thymus of mice and humans does not express myosin heavy chain proteins, thus CD4+ T cells are not negatively selected for myosin in the maturation process (40). The autoantibodies frequently associated with autoimmune myocarditis may be the result of this mechanism (40, 41). The presence of myosin antibodies has been associated with antibody-mediated rejection and development of chronic allograft vasculopathy in heart transplantation (42, 43). Three hundred single-nucleotide polymorphisms have been identified in the myosin motor domain of cardiac myosin (44), but it is not yet known whether myosin antibodies detected in the patients are donor specific. The impact of cardiac myosin antibodies on heart transplantation was confirmed by a swine minor antigen-mismatch model, in which animals with strongly binding myosin antibodies after immunization of myosin pretransplant rejected grafts acutely, while animals with low and transient binding myosin antibodies had long-term allograft survival (45). The presence of donor HLA-specific antibodies in patients with antibody-mediated rejection and cardiac allograft vasculopathy precedes the detection of myosin and vimentin antibodies (43), thus again suggesting the interplay of allo- and autoimmune responses.

## Anti-Perlecan Antibodies

Perlecan is a critical component of the endothelial basement membrane and serves as a barrier between the circulating blood and the surrounding tissue (46). Perlecan is a large, extracellular matrix proteoglycan with many functions. Perlecan can act as a coreceptor for fibroblast growth factor 2 to stimulate cell proliferation as demonstrated in a rat transplant model (47). Injection of LG3, a C-terminal fragment of perlecan, increases cell migration and accumulation of recipient-derived α smooth muscle actin-positive cells in neointima in a MHC-mismatched allogeneic aortic segment transplant mouse model (48). Elevated levels of LG3 have been found in the sera of kidney-transplant recipients with acute vascular rejection (49). Studies have shown that vascular injury prompts release of apoptotic exosome-like vesicles which trigger the production of antibodies to LG3 (50). Pre or posttransplant, higher levels of LG3 antibodies have been found to be associated with acute vascular rejection in kidney-transplant recipients (51). Anti-perlecan antibodies have been detected in the sera of transplant patients with glomerulopathy (52). Pretransplant LG3 antibodies have also been shown to be associated with an increased risk of delayed graft function. Passive transfer of LG3 antibodies can cause microvascular injury in a kidney ischemia-reperfusion injury mouse model (53).

### Other Non-HLA Antibodies

The endothelium lines the interface between the graft and recipient tissue and antigens expressed by these cells are the first line targets of the recipient’s immune system. Antibodies against four non-HLA endothelial antigens: endoglin, Fms-like tyrosine kinase-3 ligand (FLT3), EGF-like repeats and discoidin I-like domains 3 (EDIL3), and intercellular adhesion molecule 4 (ICAM4) have been identified in the sera of renal transplant patients. Endoglin is a membrane glycoprotein primarily expressed on vascular endothelium. It regulates angiogenesis and revascularization (54). FLT3 is a receptor tyrosine kinase regulating cell differentiation, survival, and proliferation (55). It is suggested that activation of FLT3 signaling promotes angiogenesis of multiple myeloma (56). EDIL3 is secreted by endothelial cells and associates with extracellular matrix. The expression of EIL3 inhibits leukocyte–endothelial adhesion (57). ICAM4, also known as the Landsteiner–Wiener blood group antigen, is a single-spanning transmembrane protein. ICAM4 mediates binding of leukocytes *via* its interaction with integrin (58). The presence of these antibodies is associated with the production of posttransplant donor-specific HLA antibodies, antibody-mediated rejection, and early transplant glomerulopathay (59). In heart transplantation, the presence of anti-endothelial cell antibodies is also associated with increased risk of early acute rejection. Nevertheless, long-term outcome and patient survival are not affected by these antibodies (60).

Renal disease can also result from autoimmune injury, such as systemic lupus erythematosus. Anti DNA/histone antibodies found in the kidney with lupus nephritis can promote pathogenesis by stimulating cytokine production and inflammation (61, 62). Since DNA/histone are only exposed after cell breakdown as happened in cell apoptosis or necrosis, these antibodies are unlikely to be the initiating autoantibodies in lupus nephritis. However, the presence of pretransplant antibodies to apoptotic cells has been shown to correlate with allograft loss in kidney transplantation (63). Mycophenolate mofetil plus corticosteroids which is commonly used in organ transplantation is the standard-of-care treatment for lupus nephritis (64).

## Conclusion

Vigorous efforts to investigate non-HLA antibodies, their impact on graft outcome, and influence on the alloimmune and autoimmune processes are still ongoing. With the availability of commercial reagents, the study of several of these antibodies to non-HLA antigens has moved to the clinical setting. As more studies populate the literature, there are common themes that appear consistently. The antibodies to these non-HLA-specific antigens do appear to be part of the overall graft dysfunction response (Table 1). These non-HLA antibodies are often checked when antibody-mediated rejection is suspected, but no donor-specific HLA antibody is detected. The presence of non-HLA antibodies may also be tested for patients who are deemed to have a high risk of antibody-mediated rejection. Commercial available Luminex bead-based reagents, which can provide detection of non-HLA antibodies in a standardized manner, may promote more transplant programs to evaluate non-HLA antibodies. Some antigens targeted by non-HLA antibodies are not polymorphic at the amino acid sequence. These antibodies may also bind the recipient’s tissue and have a global effect in the recipient as happened in patients with AT_1_R antibodies. It is unclear if other non-HLA antibodies also have a broader effect or if the effect is limited to the allograft because the graft expresses these antigens at the higher level. The presence of non-HLA antibodies may predispose patients to an increased risk to develop donor-specific HLA antibodies and rejection. Whether the antibodies to non-HLA antigens appear before the donor HLA-specific antibodies or the reverse is unknown at this time. The presence of these non-HLA antibodies may indicate underlying immune status. Lupus patients who usually display autoantibodies have higher level pro-inflammatory cytokines, which may promote immune response to allo-antigens. Autoantibodies may also cause allograft injury through complement-dependent cytotoxicity or antibody-dependent cell-mediated cytotoxicity. Donor HLA antigens released from injured cells may be captured by the recipient dendritic cells and trigger donor-specific HLA antibody production. Alternately, tissue injury caused by donor-specific HLA antibodies may expose neoantigens which lead to auto-antibody production. Certainly each scenario may be part of different overall immune responses in different organs, with different immunosuppressive regimes, and in different genetically disparate settings. The identity of some non-HLA antigens targeted by these antibodies remains elusive. Nonetheless, a continuing theme of these studies is that both alloimmune and autoimmune mechanisms impact graft outcome and may be synergistic to each other.

**Table 1:** **Non-human leukocyte antigen (HLA) antibodies in solid organ transplantation**.

Antibody	Organ	Reference	Number of patients	Major findings
Major histocompatibility complex class I chain-related gene A (MICA)	Kidney	(10)	1,910	The presence of MICA antibodies pretransplant is associated with an increased rate of graft loss
MICA	Heart	(11)	44	The prevalence of MICA antibodies is higher in patients with acute rejection. The appearance of MICA antibodies posttransplant precedes acute rejection
MICA	Heart	(12)	95	Development of MICA antibodies is associated with antibody-mediated rejection
Angiotensin II type 1 receptor (AT_1_R)	kidney	(18)	33	Out of 33 patients with refractory vascular rejection, AT_1_R antibodies detected in 16 patients with malignant hypertension, but without HLA antibodies. Passive transfer of AT_1_R antibodies induces vasculopathy and hypertension in a rat kidney-transplantation model
AT_1_R	Kidney	(65)	599	Patients with AT_1_R antibodies >10 U had a 2.6-fold higher risk of graft failure 3 years posttransplant and a 1.9-fold higher risk of acute rejection within the first 4 months posttransplant
AT_1_R	Kidney	(20)	97	The presence of strong AT_1_R antibodies (>17 U) is associated with antibody-mediated rejection, but not cell-mediated rejection
AT_1_R	Heart	(66)	200	Pretransplant AT_1_R antibodies alone are not associated with antibody-mediated rejection and cell-mediated rejection, but the presence of both AT_1_R and *de novo* donor-specific HLA antibodies increases the rate of antibody-mediated rejection and cell-mediated rejection
Endothelin-1 type A (ET_A_R)	Heart	(21)	30	Increased levels of ET_A_R and AT_1_R are associated with cell-mediated rejection and antibody-mediated rejection
Vimentin	Kidney	(37)	70	Levels of pretransplant vimentin IgG, but not IgM, are elevated in patient with interstitial fibrosis and tubular atrophy
Vimentin	Heart	(38)	109	The presence of vimentin antibodies predicts transplant-associated coronary artery disease
Myosin	Heart	(42)	72	Myosin reactive T cell or anti-myosin antibodies are associated with development of chronic allograft vasculopathy
LG3 (perlecan)	Kidney	(51)	60	Antibodies against the LG3 domain of perlecan are present in patients with acute vascular rejection
LG3 (perlecan)	Kidney	(53)	172	Pretransplant LG3 antibodies are associated with delayed graft function
Other non-HLA endothelial cell antigens	Kidney	(59)	150	The presence of antibodies against endoglin, Fms-like tyrosine kinase-3 ligand, EGF-like repeats and discoidin I-like domains 3, and intercellular adhesion molecule 4, is associated with the production of posttransplant donor-specific HLA antibodies, antibody-mediated rejection, and early transplant glomerulopathy

## Author Contributions

XZ and NLR participated in literature review and writing.

## Conflict of Interest Statement

The authors declare that the research was conducted in the absence of any commercial or financial relationships that could be construed as a potential conflict of interest. The reviewer, DW, and handling editor declared their shared affiliation, and the handling editor states that the process nevertheless met the standards of a fair and objective review.

## Abbreviations

HLA, human leukocyte antigen; MICA, major histocompatibility complex class I chain-related gene A; AT_1_R, angiotensin II type 1 receptor; ET_A_R, endothelin type A receptor; NK, natural killer; GPCR, G protein-coupled receptor; SNPs, single-nucleotide polymorphisms; PLC, phospholipase C; cAMP, cyclic adenosine monophosphate; MAPK, mitogen-activated protein kinase; ERK, extracellular signal-regulated kinase; FLT3, Fms-like tyrosine kinase-3 ligand; EDIL3, EGF-215-like repeats and discoidin I-like domains 3; ICAM4, intercellular adhesion molecule 4.
